# Supplementary material for: Are carbon emissions trading and green financial instruments synergistic? -Comprehensive quantitative research based on content analysis
Source: PLoS One. 2024 Mar 7;19(3):e0298601. doi: 10.1371/journal.pone.0298601 (PMC10919601; doi:10.1371/journal.pone.0298601)
Supplement: S1 Data — (ZIP) [file pone.0298601.s001.zip › Data/地级市Z_F工作报告.zip!/║ú─╧/╚2╤╟╩╨/└┤╫╘╣1⁄2╓┌║┼í╛┬φ┐╦╩2╛▌═°í┐_2012.docx]

各位代表：

现在，我代表市人民政府向大会作工作报告，请予审议，并请市政协各位委员和其他列席人员提出意见。

过去五年工作回顾

2007年以来，在中共三亚市委的正确领导下，在市人大、市政协的监督支持下，市政府坚持以科学发展观为指导，牢牢抓住国际旅游岛建设等重大机遇，积极应对国际金融危机等严峻考验，努力打造中外游客的度假天堂和三亚人民的幸福家园，胜利完成了市五届人大一次会议确定的主要目标和任务，三亚发展已步入了一个全新的时期。

一、全市国民经济高速增长，综合实力迈上新台阶

五年来，全市生产总值年均增长17.9%（按可比价格计算，下同），2011年预计达到286亿元（不含农垦，未经省统计局评估，下同）。全社会固定资产投资年均增长41.2%，2011年达到355亿元，五年来累计完成1136亿元。社会消费品零售总额年均增长25.8%，2011年达到80亿元。发展质量逐步提升，全市人均生产总值年均增长15.2%，2011年达到56500元（按户籍人口计算）。地方财政一般预算收入年均增长38.6%，2011年达到50亿元，其中税收收入44.6亿元。金融形势保持稳定，截至去年11月底，全市金融机构本外币各项存款余额582.6亿元、贷款余额263.2亿元，分别达到2006年底的3.6倍和3.5倍，不良贷款率从2006年底的19.57%下降为0.62%。

二、产业结构调整有序实施，生态环保取得新成效

五年来，我们着力提升旅游产业发展质量，逐步调整房地产业内部结构，加快农业产业化进程，积极培育海洋、智慧、商贸、文体等产业，亚龙湾等度假区设施日益完善，海棠湾“国家海岸”等区域开发快速推进，创意产业园等园区建设稳步开展，各产业载体项目总体上顺利实施，三次产业结构从22.8:23.6:53.6转变为12.1：21.2：66.7。2011年，全市接待过夜游客已从2006年的454.9万人次增长到1000万人次，旅游总收入从65.4亿元增长到157亿元；设施大棚种植面积从0.6万亩发展到3.5万亩，冬季瓜菜种植面积从14万亩发展到17万亩；2011年，全市海洋经济总产值达到120亿元，五年来年均增长18.5%；中兴通讯等项目稳步推进，市高新技术产业园等园区五年来累计完成工业总产值43.9亿元；市内免税店建成营业，海棠湾国际购物中心项目正式启动，购物退免税政策有序实施；完成了文化体制改革“转企改制”，积极引进文化企业和项目，成功举办了第57届、第60届世界小姐总决赛等国际赛事与活动。五年来，我们认真开展节能减排工作，亚龙湾冰蓄冷区域供冷站已开始发挥节能效益，生活垃圾无害化处理率和城镇生活污水集中处理率分别达到100%和81.7%。积极推广节能环保天然气公交车、绿色照明和太阳能热水系统建筑应用，实施民用建筑节能评估审查，拆除了华盛天涯水泥厂，在全省率先拆除了全部粘土实心砖生产线，循环农业沼气用户已突破2万户。认真实施生态市建设规划，推进三亚湾生态环境修复和保护，完成了沿海基干林带恢复建设工程，有效开展造林绿化等工作，全市森林覆盖率达到68%。

三、城乡建设步伐继续加快，统筹发展取得新进展

坚持规划先行，先后完成了三亚市土地利用总体规划、新一轮城市总规以及城市综合交通规划等专项规划编制，完成了海棠湾等重点片区控规、月川和阳光海岸等部分区域旧城改造规划以及新农村规划设计。五年来，凤凰机场二期扩建、东环铁路三亚站、亚龙湾站、南山货运港一期工程等重大基础设施已建成启用，凤凰岛国际邮轮港累计接待出入境旅客超过40万人次，2011年凤凰机场旅客吞吐量已突破1000万人次。绕城高速公路即将投入使用，西线高速公路至天涯海角道路、吉阳大道、鹿回头海景大道建设以及河西路等市区道路改造已完成，新风桥重建等工程有序推进，城乡电网、供水、供气以及城市无障碍设施建设稳步开展。大力发展公共交通，增设公交线路，增加和更新公交车辆，加强了道路交通环境综合整治。启动数字化城管系统和数字三亚地理空间框架建设，积极开展“民生体验”和全民参与城市管理活动，将部分管理权限委托给区镇，强化了属地管理。广泛开展爱国卫生运动，成为我省首个国家卫生城市。大力实施“铁锤行动”，拆除违法建筑180多万平方米，打击遏制了违法建设行为。五年来，以文明生态村建设为载体，统筹推进农村经济发展、基础设施建设和公共服务均等化，改善了农村生产生活条件。目前，全市文明生态村覆盖率已达到80％。基本建成了互联互通的农村公路网络体系，在全省率先实现行政村主村道100%硬板化。大隆水利枢纽工程投入运行，病险水库除险加固稳步实施，农业科技110服务在两区六镇和农场实现全覆盖，扶贫开发等工作取得新成效。

四、改革开放事业深入推进，三亚发展焕发新活力

探索实施服务业综合改革，被确定为全国首批37个服务业综合改革试点区域之一。着力推进市属国有企业调整重组，积极建立现代企业制度，坚持实施中小企业成长工程，鼓励、引导行业协会和中介组织发展，市场经济环境进一步优化。强化品牌保护意识，2007年以来引导市场主体成功申请商标注册717件，并在“海棠湾”商标维权工作中取得突破性进展。稳步推进农村土地产权制度改革试点，全面完成了集体林权制度改革主体任务。积极完善农村金融服务，三亚金凤凰村镇银行、崖城镇众树农村资金互助社已开业。在全省率先实施国家基本药物制度，公立医院改革试点和基层医疗卫生体制综合改革稳步推进。认真实施政府收支分类改革，完善预算支出标准体系，推行财政预算项目及资金安排听证制度。不断优化财政支出结构，强化了支出绩效评价管理。以2008年、2011年在三亚举行的重大国事活动为契机，积极促进对外交流，充分利用上海世博会海南周活动、我省历次香港招商活动和“珠洽会”、“厦交会”等平台，拓展合作空间，取得积极成效。五年来，全市实际利用外资8.9亿美元。目前，进驻三亚的国际品牌酒店已达到48家，凤凰机场国际航线和港澳台航线已达到38条。

五、人民生活质量大幅提升，发展惠民实现新突破

加大民生工程投入力度，确保每年市财政新增财力的一半以上用于改善民生。五年来，全市城镇居民人均可支配收入和农民人均纯收入年均分别增长16.3%和15.3%，2011年已达到20068元和7580元；全市累计新增城镇就业岗位近12万个，实现农村富余劳动力转移就业6万多人，城镇登记失业率控制在2%左右；在全省率先实施城乡居民基本医疗保障一体化，初步建立了覆盖城乡居民的基本养老保险制度，低保标准逐年提高；着力推进“菜篮子”工程建设，全市常年标准化“菜篮子”基地已超过1.5万亩，设立了6家平价蔬菜超市，建立了物价上涨财政联动补贴机制；2008年以来已开工建设保障性住房22942套，竣工7381套，完成分配5135户，改造农村危房近3000间，全市茅草房改造任务也已完成。五年来，积极推进中小学布局调整和规划建设，建成了三亚中等职业技术学校，大力支持琼州学院三亚校区等高等院校建设发展，困难学生资助体系进一步健全；市中医院新区、市人民医院改扩建一期项目建成投入使用，解放军总医院海南分院已开始试运行，社区卫生服务网络和行政村卫生室覆盖率达到100%；加快了社区数字电影院、“24小时自助图书馆”等设施建设，市体育中心已投入使用，城乡文化体育活动蓬勃开展。五年来，社会治安环境明显改善，在2009年荣获“长安杯”；食品药品安全保障有力，信访、安全生产等工作进一步强化；加强应急联动机制和队伍建设，有效应对了台风、强降雨等自然灾害和突发事件。积极开展国防动员、民兵预备役工作，两次被评为“全国双拥模范城”。人口计生、民族宗教、新闻、统计、档案、修志、地震、人防等工作扎实推进，红十字、妇女儿童、老龄和残疾人事业蓬勃发展。

六、政府自身建设不断强化，行政能力达到新水平

市政府政务服务中心从2010年10月起正式运行，截至去年底共有770多项行政审批（服务）及社会公共服务项目进驻，审批和服务行为进一步规范，效率显著提高。对海棠湾管委会机构进行了调整，逐步授予海棠湾市一级行政审批权限，并在此基础上积极探索推进强镇（区）扩权改革。着力强化政府工作督查，出台了《三亚市行政机关效能监察实施办法》，认真开展政府绩效考评，切实落实各项问责制度，行政效能进一步提高。认真执行市人大及其常委会的决议、决定，自觉接受市人大和市政协的监督，虚心听取各界人士的建议、意见。五年来累计办复市人大代表建议、批评和意见397件、市政协委员提案572件。积极拓展政务公开的领域和内容，不断完善和严格落实重要岗位、重点领域权力运行制度，实行重大项目全程监督，实现全市村集体“三资”全面委托。加大审计、监察工作力度，严肃查处各类违法违纪案件，政府廉政建设进一步加强。

各位代表，回顾过去，我们深深地感到，三亚发展所取得的一切成绩，都离不开省委、省政府和市委的正确领导，离不开市人大、市政协、各民主党派、工商联、人民团体的监督支持，离不开中央和省驻三亚各单位、驻三亚部队、武警官兵、公安司法干警的参与配合，离不开海内外广大朋友的关心帮助，是全市各族人民不断开拓创新、攻坚克难、团结奋斗的结果。在此，我谨代表市政府，向所有为三亚的建设发展事业付出辛勤劳动，给予帮助支持的同志们、朋友们，表示衷心的感谢，并致以崇高的敬意！

在总结成绩的同时，我们也清醒地看到，我市经济社会发展中仍然存在不少矛盾和问题，政府工作中仍然存在一些不足之处，主要表现在：产业结构相对单一，高增值产业比重过小，尚未形成强劲的经济增长点；旅游产品的国际竞争力还不够强，旅游要素的国际化改造有待加快；城镇化的加速推进给基础设施带来不小的压力，城市功能与国际旅游岛建设的要求差距较大，旧城改造和设施配套亟需加快，城市管理的技术手段和常态化机制有待完善；教育、医疗、文化等社会事业基础薄弱，供需矛盾较为突出；在物价上涨的压力下，城乡居民增收效果受到一定程度的影响；随着改革发展的不断深入，各种新问题逐步显现，社会管理亟待加强；政府执行力仍需提高，基层机构和组织的管理职能尚未得到充分发挥。对于这些问题，我们将高度重视，并采取有效措施在今后的工作中努力加以解决。

今后五年奋斗目标

今后五年是我市全面完成“十二五”规划，加快推进世界级热带滨海度假旅游城市建设的关键时期，政府工作的总体要求是：以邓小平理论和“三个代表”重要思想为指导，深入贯彻落实科学发展观，紧紧围绕国际旅游岛建设，加快优化产业结构，着力转变发展方式，积极打造生态文明，不断深化改革开放，统筹城乡建设发展，全力改善人民生活，加强和创新社会管理，努力建设更加卓越、更加和谐的三亚，在全省率先实现全面小康。

根据市第六次党代会作出的部署，新一届政府要紧紧围绕“六个更加”的目标任务，抓好以下几个方面的重要工作：

一、提升产业实力，实现“经济更加活跃”的目标

着力推进旅游要素国际化改造，增强旅游核心竞争力，继续推进房地产业、现代农业、智慧产业、商贸产业、海洋产业以及文化体育产业发展，积极培育金融产业和总部经济，实现产业适度多元化，推动经济健康较快发展。力争今后五年年均经济增长率达到14%左右，到2016年全市生产总值突破500亿元，其中服务业增加值比重达到70%以上；全社会固定资产投资达到560亿元；社会消费品零售总额达到210亿元。

二、深化改革开放，实现“城市更加知名”的目标

依托国际旅游岛建设和国家服务业综合改革试点，积极推进体制机制创新，加快形成更加符合国际惯例的体制机制和活动方式。着力完善旅游管理体制和运行机制，放宽旅游市场准入，在出入境政策、特色旅游项目、企业上市融资等方面开展先行先试。继续深化市场经济体制改革、行政管理体制改革，积极开展多领域国际交流与合作，进一步推动与泛珠三角等区域和周边市县的合作。

三、统筹城乡发展，实现“城乡更加融合”的目标

按照新一轮城市总规，科学推进城镇体系建设，着力完善配套基础设施和功能，加快城镇化进程，力争2016年全市城镇化率达到70%。分类实施村庄整治，积极推进农村道路、供电、供水、通讯、沼气以及教育、卫生、文体等设施建设，完善农村公共服务。建立健全城乡统一的市场网络，深化农村综合配套改革，引导土地、资本、技术、劳动力等生产要素合理流动。

四、创新社会管理，实现“社会更加文明”的目标

加强和创新社会管理，着力发挥基层管理作用，强化各区镇的社会管理和公共服务职能，引导基层自治组织、社会团体、行业协会和其它非政府组织提供公共服务，参与社会管理。建立数字化城管系统，引进国内外先进管理理念和模式，提高城市管理现代化水平。积极开展三亚“文明大行动”，努力提升城乡文明程度和公民文明素质，力争2014年成为“全国文明城市”。

五、加强生态建设，实现“生态更加宜人”的目标

大力发展低碳产业，推广太阳能等能源利用，努力提高资源综合利用效率。积极完善硬件设施和管理制度，确保实现节能减排工作目标。有针对性地强化北部低山丘陵、中部台地平原以及滨海地区三大生态功能区建设，实施海洋生态综合管理，开展河流整治、湿地保护、造林绿化、环境综合治理等工作，加大生态恢复和保护力度，促进人与自然和谐发展。

六、实施民生规划，实现“人民更加幸福”的目标

坚持以人为本，把改善民生作为政府工作的根本目标，在今后五年内投入财政资金365亿元，即总体综合财力的一半以上，用于实施《三亚市2012―2016年重点民生项目发展规划》。要以教育、就业、公共卫生及基本医疗体系建设、社会保障、住房保障、公益性文化体育建设、城乡居民增收、生态环境保护、公共安全建设和社会主义新农村建设等十大民生工程40个重点项目为载体，完成改善民生的总体目标。特别是要在全省率先实现城乡居民基本医疗保险统一标准全覆盖，率先系统地建立健全失地农民保障机制，力争实现城乡居民收入倍增，保持保障性住房和城乡居民危房改造投入水平全省领先，使全市教育发展条件显著改善，就业再就业及创业扶持效果显著改善，把三亚建设成为全市人民的幸福家园。

今年主要任务

今年是全面贯彻市第六次党代会战略部署的第一年，实现新一届政府五年任期目标，首先要做好今年的工作。尽管今年的宏观经济形势可能更加复杂严峻，但从总体上看，我省仍然处于重要的战略机遇期和发展上升期，国际旅游岛建设的政策效应持续释放，对三亚发展仍然有利。从我市自身的情况看，以旅游为龙头的现代服务业、热带特色现代农业以及各项新兴产业属于政策鼓励和扶持的范围，城市区位、资源环境和利好政策的综合优势对国内外众多企业具有较强的吸引力，支撑发展的积极因素仍然不少。我们一定要坚持稳中求进的总基调，紧紧围绕发展第一要义，牢牢把握工作主动权，准确判断形势，充分利用机遇，灵活应对挑战，千方百计推动经济社会平稳较快发展。综合考虑各种因素，建议今年经济社会发展的主要预期目标为：全市生产总值增长12%以上，全社会固定资产投资增长20%以上，社会消费品零售总额增长20%，地方财政一般预算收入增长15%，全市接待过夜游客增长10%，城镇居民人均可支配收入增长12%，农民人均纯收入增长12%，居民消费价格总水平涨幅控制在4.5%左右，城镇登记失业率控制在3%以内，完成省下达的节能减排任务指标。为实现上述目标，今年要努力抓好以下几个方面的工作：

一、加快优化产业结构，构建特色体系，增强经济实力

产业是经济的支撑。要坚定不移地实施产业结构调整，不断提升传统优势产业发展质量，积极培育新兴产业、高增值产业，建立结构更加合理的产业体系。

（一）提升旅游产业发展质量。认真落实旅游发展规划布局，着力增强主城区的旅游服务中心功能，积极推进海棠湾“国家海岸”、亚龙湾二期、南山旅游区、红塘湾旅游度假区等片区建设。加快国际邮轮港建设，吸引邮轮入驻，争取国家有关部门支持，发展海上邮轮边境旅游。加快推进三亚河口游艇港规划建设和东锣、西鼓无居民海岛开发，规范发展海上旅游观光。借助沃尔沃环球帆船赛等一流赛事，完善基础设施，开发海洋体育旅游项目。继续推进亚龙湾热带天堂森林公园建设，加快半岭温泉旅游度假区等项目进度，着力培育和发展康复疗养旅游。积极推进旅游服务标准化和国际质量认证，进一步完善旅游公共服务体系。继续开展旅游环境综合整治，建立健全旅游管理长效机制。创新旅游营销方式，增强营销的针对性，提高营销的水平和效率。

（二）保持房地产业平稳健康发展。引导企业产品转型升级，鼓励有实力、有信誉的企业发展星级宾馆、度假村等项目，积极探索和规范推进分时度假酒店、产权式酒店、酒店式公寓等项目开发管理，规范家庭旅馆经营和房屋租赁经营，加大商业地产和商务办公地产发展力度。优化产业布局，严格控制滨海一线住宅建设。在旧城改造过程中，除回迁安置需要外，限制新建普通住宅。着力加强房地产项目配套服务设施建设，促进社区服务产业化发展。

（三）进一步推动现代农业发展。继续推行“企业+合作社+基地+农户”的模式，加快建设三亚南果冬季瓜菜设施农业示范基地等项目，力争全市推广设施栽培瓜果菜面积4万亩。引导企业和农民专业合作社建立标准化基地和现代化加工配送体系，扩大农产品加工业规模。大力扶持热带水果、热带花卉、冬季瓜菜、南繁育制种等特色产业发展，积极推进畜禽养殖小区示范点和种苗繁育体系建设。建立健全三级畜牧兽医防疫体系，进一步完善四级农产品质量安全检验检测体系，确保农产品、畜产品安全。

（四）打造智慧产业增长点。继续完善创意产业园基础设施，着力加快中兴通讯三亚研发中心等项目建设，积极推进用友三亚软件产业园、易视芯芯片研发基地等项目，进一步吸引有实力的知名企业入驻。加快海南省动漫产业基地、惠普云计算与3D动漫产业园建设，促进动漫产品及其衍生品的开发生产。积极发展电器装备与配送、物流配送等配套服务项目。

（五）挖掘海洋产业发展潜力。在促进滨海旅游业发展的同时，加快崖州中心渔港建设，继续实施渔业综合补给船项目，淘汰小旧渔船，开发外海渔场，建设南海渔业补给基地。依托海洋牧场继续发展休闲渔业。加强渔业生产安全服务体系建设，建立健全水产品质量安全监管体系。积极发展贝类养殖，培育水产品加工龙头企业，加大出口力度。加强海洋科技研究，发展热带海洋生物工程和海洋能源利用等新兴产业。

（六）继续实施“文化强市”战略。深入推进文化体制改革，鼓励非国有资本投资兴办文化企业。积极开发传承黎、苗、回等少数民族文化，加快推进崖城国家历史文化名镇和保平国家历史文化名村保护与整治、中国衣食父母园等项目，打造本地特色文化产品。加强知识产权保护，为文化工作者提供良好的策划、创作、生产条件，吸引外来人才。积极推进天涯不夜城、南中国海影视文化生态园、海棠湾梦幻娱乐不夜城等项目，引进和培育品牌会展。

（七）大力发展商贸产业。争取进一步放大购物退免税政策的效应，积极推进海棠湾国际购物中心、亚龙湾国际品牌直销购物中心和凤凰、吉阳旅游商业街等项目建设。继续实施农贸市场升级改造，加快推进三亚市凤凰产地集配中心、吉阳农产品批发市场、崖城果蔬储运销售中心等项目。规划建设相对集中的家居装饰市场和建筑材料市场，积极开展家政服务体系建设试点，不断完善农村流通网络和服务体系。

（八）积极培育金融产业和总部经济。不断完善各项具体措施，落实《三亚市培育促进总部经济发展暂行规定》、《三亚市支持金融业发展若干政策》，鼓励各类国际性、全国性、大区域性总部和职能性总部企业入驻三亚，鼓励各类金融机构总部和地区总部入驻三亚或在三亚设立分支机构。加快三亚南航区域总部大厦建设，积极支持阳光金融广场等项目。充分利用现有金融资源，提高银行存贷比。探索开展离岸金融等业务试点，稳步实施社会信用体系建设，切实加强金融风险监测评估。

二、加快统筹城乡发展，优化空间结构，筑牢发展基础

要在新一轮城市总规的指导下，积极打造城市中心，完善基础设施和功能，优化市域空间结构，促进城乡一体化建设，为长远发展打下基础。

（一）大力推进中心城区建设发展。全面开展三亚湾城市活力中心规划编制工作，尽早实现中心城区控规全覆盖。根据控规要求，制定、完善设计方案，加快实施阳光海岸、月川等旧城改造，推动商业、金融、商务办公、文化娱乐以及旅游服务等设施和功能向三亚湾城市活力中心集聚，有选择地推动各类设施和功能向河西三亚湾旅游服务中心、月川城市服务中心等区域集聚，打造现代化、集约化的城市核心区。加快推进鹿回头等区域开发和南边海、下洋田、榕根、东岸村等旧城改造，促进核心区外围片区和组团实现特色化发展。

（二）着力完善基础设施。加快编制市政基础设施专项规划。积极推进凤凰机场三期扩建和高铁三亚站至凤凰机场段建设，加快实施“三港分离”，完善绕城高速公路连接线和各镇道路交通基础设施。完成新风桥重建，继续推进全市社区道路改造、月川市政道路建设等工程，推进停车场规划建设，尽早实施三亚大桥重建、月明桥建设等项目。积极构建慢行交通系统，进一步完善无障碍通道和设施。加快城镇供水工程建设和管网扩建，继续推进天然气输配管网和城乡电网建设。分区分级配建教育、医疗、文体、商业、金融、邮电以及行政管理和社会服务等各项设施，加快打造“半小时便利生活服务圈”。合理利用土地，避免重复建设，推动城市立体化发展，强化地下空间利用。

（三）进一步提升城市管理水平。加快数字化城市监控系统、管理系统和数字三亚地理空间框架建设。继续开展全民参与城市管理活动，进一步明确区镇在城市管理中的主体责任，合理下放管理权限。继续开展“铁锤行动”，力争在年内基本完成消除违建存量的任务。积极引导城乡居民依法报建，强化属地管理，发挥村（居）委会和基层组织的作用，加强执法巡查，严密监控新增违建。增加公交运力，优化公交线路，提升公交便捷程度和服务水平，鼓励公交出行。积极推进三亚市智能交通系统建设，继续开展道路交通环境综合整治，实施“文明交通行动计划”。加快完善垃圾收集、中转、处理体系，积极开展垃圾分类收集试点。认真实施三亚市国家卫生城市长效管理机制和细则，对重点路段和区域实行全天候网格化巡查管理，进一步优化城市环境，迎接国家卫生城市的复检。

（四）积极推进特色城镇和新农村建设。加强村镇规划建设管理，推动吉阳镇和凤凰镇与中心城区一体化发展，突出海棠湾、崖城、天涯、育才等各镇特色，完善全市城镇体系。以文明生态村建设为载体，继续改善农村生产生活条件。全力推进大隆水库灌渠工程建设，积极实施病险水库除险加固、河道治理和田洋整治，在保障农业用水的同时，筑牢防汛安全基础。着力推进农村公路通畅工程和桥梁改建等项目建设，着手开展现有农村公路拓宽和升级改造工作。加快完成育才镇农村集中供水工程，逐步建立农村安全饮水项目长效管理机制。继续完善农村公共文化设施，推进多功能服务中心、农家书屋、文化室、篮球场以及休闲广场建设。进一步加大扶贫工作力度，重点实施整村推进，着力加强扶贫攻坚。坚持开发式扶贫方针，扩大产业化扶贫覆盖面，增强扶贫对象的自我发展能力。

三、加快实施民生规划，改善人民生活，确保发展惠民

改善民生是发展的根本目的。要加大投入力度，认真实施重点民生项目发展规划，不断提升人民群众生活水平。

（一）努力促进城乡居民增收。加快完善财政供养人员工资及津补贴制度，完善企业工资支付保障机制，逐步推行集体工资协商制度，进一步提高最低工资标准。积极引导乡镇企业发展，规范开发休闲农业、乡村旅游等项目，拓宽农民增收渠道。切实落实“三农”各项补贴、石油价格补贴等支民惠民补贴。新建常年标准化“菜篮子”基地3000亩，继续推进平价蔬菜超市和农贸市场平价蔬菜摊位建设，加强市场价格监控，落实物价上涨财政联动补贴机制，减弱物价因素对居民增收的影响。

（二）进一步加强就业工作。着力完善公共就业服务体系，加快公共就业信息化进程，加强社区、行政村就业创业服务平台建设。积极鼓励各单位开发公益性岗位。抓好高校毕业生、城镇下岗失业人员以及其他扶持对象的就业培训、服务和指导，继续实施农村劳动力转移培训阳光工程。加快创业孵化基地、实训基地配套建设，积极开展小额担保贷款业务，提供创业项目支持、开业指导和跟踪服务，以创业带动就业。

（三）不断完善社会保障体系。继续推进城乡居民基本养老保险工作，提高基础养老金发放标准。巩固城乡居民基本医疗保障一体化成果，进一步完善定点医疗机构准入制度和考核指标体系，规范医疗服务行为，探索区域协作和异地就医新模式。逐步提高各项社会保险统筹层次，扩大基金调剂范围，增强基金抗风险能力。探索启用“社会保障卡”，构建“一号一卡一网”的信息网络体系。不断完善城乡低保标准动态增长机制，积极开展社会救济和社会福利工作。

（四）强化保障性住房建设和管理。大力推进“同心家园”等保障性住房项目建设，力争在今年内竣工10464套，其中经济适用住房4203套、限价商品房5689套、公共租赁房321套、棚户区改造安置房251套。此外，要新开工建设保障性住房6699套，完成农村危房改造800套。抓好保障性住房审核分配，加强资格审查，建立健全保障性住房准入和退出机制，确保公开、公平、公正分配和合理使用。

（五）大力发展教育事业。推动教育投入与经济同步增长，特别是加大农村教育投入，加强农村中小学建设，促进义务教育均衡发展。继续推进中小学布局调整，抓好校舍建设，完善教学设施。深入实施校长引培工程，加大教师培训力度，提高教育教学质量。加快推进区镇中心幼儿园和分园建设，扶持、规范民办幼儿园办园行为，在普及学前教育的基础上，积极创建示范性幼儿园。加快普及高中阶段教育，提高中等职业技术学校办学水平，面向市场需求建设一批实训基地和重点专业，促进校企合作。继续推进三亚学院和琼州学院三亚校区等院校建设，加快打造科技创新、科技成果转化平台。不断完善各阶段教育资助政策，切实促进教育公平。

（六）提高医疗卫生服务水平。加快推进市人民医院改扩建二期工程、市中医院三期工程和农垦医院改造工程，实施市妇幼保健院整体搬迁项目。继续实施基本药物制度，加快公立医院改革步伐。进一步加强城市社区和农村卫生服务网络建设，继续推进基层医疗卫生体制综合改革。积极发挥解放军总医院海南分院的帮带作用，加大市级医疗卫生机构对农村卫生院的对口帮扶力度，继续探索推行基层首诊、分级医疗、双向转诊模式。全面加强公共卫生服务体系建设，进一步促进公共卫生服务项目均等化。继续强化药品、医疗器械和餐饮食品、保健食品、化妆品安全监管工作。

（七）丰富群众文化体育生活。积极构建公共文化服务体系，开工建设市群众艺术馆，尽早完成市图书馆改造，新建12个“24小时自助图书馆”。深入开展群众性文化活动，坚持送文艺演出、送电影、送图书下乡。认真开展“扫黄打非”专项整治，净化社会文化环境。充分发挥市体育中心的功能和社会效益，进一步完善公园、社区等场所的体育设施，加快推进各区镇全民健身体育广场规划建设，大力开展群众性体育活动。

（八）加强和创新社会管理。积极开展三亚“文明大行动”，大力加强精神文明建设，深入推进法制宣传教育。强化政府社会管理职能，引导各类社会组织增强社会服务能力，支持人民团体参与社会管理和公共服务。加强村（居）委会建设，强化城乡社区自治和服务功能。坚持实施入户走访计划，做好暂住人口、出租房屋登记、办证工作，完善流动人口管理体系。加强国际警务交流与合作，提高境外人员管理和服务水平。进一步完善社会治安防控体系，围绕突出治安问题开展打击整治行动。严格落实“属地管理、分级负责”和“谁主管、谁负责”的信访工作责任制，引导群众依法有序信访，促进问题依法及时解决。严格落实安全生产责任制，着力加强事故多发行业和领域专项整治。加快实施城镇防洪防潮工程和红树林公园地震应急避难场所建设，开展应急行动演练，增强防灾减灾和应急救援能力。大力支持国防建设，抓好国防动员、民兵预备役和人防工作，广泛开展双拥共建活动。继续做好人口计生、民族宗教、外事侨务、新闻、统计、档案、修志等工作，推动红十字、妇女儿童、老龄和残疾人事业取得新进展。

四、加快推进改革开放，破解发展瓶颈，增强发展活力

在国际旅游岛建设背景下，积极推进重点领域和关键环节改革，提高对外开放水平，为经济社会发展提供体制机制保障。

（一）围绕项目建设完善各项保障机制。产业发展、城乡建设和民生改善，都离不开项目的支撑。要围绕我省“项目建设年”的各项要求，全力抓好项目工作，扩大投资规模，做到竣工投产一批、快速推进一批、开工建设一批、谋划储备一批。一是继续深化行政审批制度改革。进一步清理、取消和调整行政审批事项，推行审批目录化管理，规范审批行为。对已取消和调整的审批事项，防止以备案、核准等名义进行变相审批；对保留的审批事项，继续推行“三集中”改革，依托市政府政务服务中心，规范流程，简化环节，提高效率，限时办结。确保审批公开、透明运行，强化监督检查，坚决查处该批不批、无故拖延、吃拿卡要和越权审批等行为。与此同时，要把好项目审批关，增强审批的区分度，让优秀的企业和项目脱颖而出、顺利进驻。二是完善项目管理制度。把全市项目分解到各区镇，并将有关工作纳入区镇发展指标考核体系，充分发挥区镇在项目管理方面的主体作用，以项目促进区镇发展，以区镇支撑全市发展。按照“一个项目、一个分管领导、一个责任单位、一个工作班子、一个倒排工期计划、一竿子抓到底”的“六个一”责任模式，实行重点项目跟踪服务，促进项目按照时间节点扎实推进。大力强化项目前期工作管理，特别是要增强政府投资项目设计、概算编制的科学性和规范性，严格限制在建设过程中变更设计、调整概算，把好决算审计关。不断完善项目招投标体制，全面考量代建、设计、施工投标者的实力和信誉，进行合理筛选，并强化合同管理和责任追究。三是进一步改进征地拆迁和用地保障机制。按照《三亚市集体土地征收补偿安置管理规定》，提高征地补偿标准。对安置区建设，要从土地、规划、资金、审批等方面予以优先安排，全力加快实施。在风情小镇、公寓小区等安置方式选择上，要尊重拆迁户自主权，并通过配建出租屋、商业房等方式使群众长期受益。着力推进创意产业园等项目安置区建设，让拆迁户真正得到实惠，在全市发挥示范带动作用。坚决打击征地拆迁安置工作中的腐败行为，切实保障拆迁户的合法权益，将保障发展用地和改善群众居住条件更好地结合起来。继续加强土地储备工作，实施市、镇（区）两级储备、收益分享。结合旧城改造、城边村改造、新农村建设等工作，推进土地整理。四是规范资金筹措机制。积极争取中央和上级资金支持。进一步加强城投公司等市政府融资平台公司管理，把好融资项目关，合理举债，增强融资能力，防范债务风险。积极配合国家开发银行融资要求，合理使用已落实的开行贷款。在大力促进产业发展，推进财源建设的同时，强化依法治税，加强收入组织和分析，挖掘增收潜力，确保财政收入持续稳定增长，增强政府基本建设能力。充分发挥政府投资尤其是基础设施项目投资的效益，推动相关社会项目和投资迅速跟进。

（二）积极推进农村综合配套改革。全面完成农村宅基地登记发证工作，进一步实施农村集体土地细分确权。在此基础上，建立规范有序的集体土地使用、管理体制，打击非法买卖集体土地的行为。加强农村土地流转指导、协调、服务和管理，强化集体经济组织或新型村民自治组织的作用，让村民在发展中把握更多的主动权，继续推进农村土地产权制度改革试点，实施城中村和城边村改造。全面推进林改综合配套改革，加强林业社会化服务体系建设。鼓励新设村镇银行、贷款公司和农村资金互助社，继续深化农村信用社改革，不断完善小额信贷、农户联保贷款和农业保险等金融服务。

（三）进一步深化市场经济体制改革。继续推进市属国有企业调整重组，抓好改制、关闭退出企业的资产处置和职工安置。推动国有资本向优势行业和重点领域集中，整合优质资源，培育龙头企业。积极推进国有企业股份制改造，力争尽快实现我市国企上市“零的突破”。支持和引导非公有制经济发展，继续实施中小企业成长工程，放宽市场准入条件，鼓励各类资本发起设立中小企业信用担保机构和融资性担保机构。进一步促进和规范行业协会、中介组织发展。

（四）加快服务业管理体制改革。根据《三亚市开展服务业综合改革试点工作总体实施方案》，细化推进各行业、各部门改革试点。在旅游方面，要继续推动政府旅游管理职能向规划编制、标准制定、行业准入和统筹协调等方面转变，完善旅游行业协会体系，突出协会管理作用。结合旅游行业创先争优活动，加强旅游企业自律管理，推动企业订立诚信公约。鼓励各种所有制企业依法投资旅游产业，着力培育具有较强竞争力的大型旅游企业集团。推进旅行社体制改革，鼓励大型旅行社拥有旅游交通等要素资源，改革完善导游等级制度。

（五）继续实施“开放带动”战略。努力提升招商引资质量，坚持“大企业进入，大项目带动”，围绕智慧产业、节能环保产业、高品质旅游和其它现代服务业等领域，按照完善控规、储备土地、策划项目、跟踪推进的流程，提供“一条龙”的高效服务，强化以商引商、产业链招商，逐步推行项目和用地捆绑招商。积极组织参加第八届“珠洽会”、第十六届中国国际投资贸易洽谈会等活动，逐步建立招商引资网络信息平台，开展网上招商。建立健全招商引资项目监督和退出机制。继续实施科技兴贸战略，围绕水产和热带花卉等优势产品，加强品牌培育，着力引进关联度高、市场潜力大、竞争力强的大型企业。转变外贸发展方式，扩大服务贸易进出口。进一步完善口岸基础设施，加快电子口岸建设，实现海空港口岸资源互联互通。

五、加快建设“两型社会”，推行低碳发展，打造生态文明

积极转变发展方式，加强节能减排、生态环保工作，在加快发展的同时保护好生态环境，实现经济社会可持续发展。

（一）积极建设资源节约型社会。强化土地开发监管，集中力量对批而未供、供而未用的土地进行处置，盘活存量土地，提升土地利用集约化水平。进一步规范海域使用权公开招标拍卖，强化海域资源管理。加快城市中水回用工程建设，推广节水技术，提高水资源综合利用效率。加强技术改造，降低供电损耗。认真实施差别电价、差别水价等措施，运用经济杠杆促进节约。加快再生资源回收利用体系建设，促进废旧资源循环利用。加强固定资产投资项目节能评估和审查。继续扩大亚龙湾冰蓄冷区域供冷站应用范围，启动酒店“节能战略”，推广建筑节能、照明节能，完善太阳能热水系统建筑应用项目管理，着力打造可再生能源建筑应用示范城市。继续推广农村地区沼气利用，新建“一池三改”户用沼气800户。

（二）积极建设环境友好型社会。加快推进污水处理工程建设、市区污水管网改造和各镇污水管网配套。认真开展污染减排单位巡查，全面实施重点污染源在线监控。强化建设项目环境影响评价审批，严格执行“三同时”制度和项目竣工环境保护验收制度。加强三亚渔港海洋环境监督，抓好重点海湾海洋环境和海岸工程监管，加快鹿回头湾岸滩整治修复和三亚湾东部岸滩人工补沙工程建设。继续推进生态市建设，实施河流生态整治、湿地保护和城市林荫工程，进一步加强饮用水水源地环境监控和保护。积极开展“绿化宝岛”行动，认真制定年度计划，扎实做好造林绿化、森林资源保护、森林生态补偿试点等工作。加强汽车尾气、城市噪声等污染治理。

加强政府自身建设

2012年是新一届政府履行职责的第一年。今后五年，市政府肩负着完成“十二五”规划各项目标，推动国际旅游城市建设的重要任务。我们必须努力适应形势需求，不断加强政府自身建设，解放思想，积极创新，真抓实干，推动三亚科学发展。

一、进一步树立责任意识，勇于担当抓发展

三亚升格为地级市以来，历届政府在市委的正确领导下，在市人大、市政协的监督支持下，牢牢抓住海南建省办特区等重大机遇，与全市人民共同奋斗，为我们打下了坚实的基础。现在，面对国际旅游岛建设这样难得的机遇，新一届政府一定要倍加珍惜，勇于担当为政之责，在过去的基础上，坚持艰苦奋斗，不断追求卓越，认真落实市委、市人大的各项决策部署，千方百计推动三亚又好又快发展。

二、进一步树立创新意识，解放思想增活力

建设国际旅游岛没有现成经验可循，需要我们结合形势的发展变化，与时俱进，改革创新。新一届政府一定要解放思想，开阔眼界，在国际旅游岛政策框架内，通过体制机制创新，突破制约发展的瓶颈；要善于学习，勤于思考，在强化理论素养的同时，借鉴先进地区的发展经验；要勇于探索，大胆实践，形成鼓励创新，宽容失误，拒绝平庸，惩治腐败的为政环境。

三、进一步树立法制意识，依法行政严要求

新一届政府要严格按照法定权限和程序履行职责、行使权力，把依法行政贯穿于工作全过程。坚持科学决策、民主决策、依法决策，不断完善和认真执行重大决策听证、财政预算听证、重要事项公示等制度。认真执行市人大及其常委会的决议、决定，自觉接受市人大的法律监督、工作监督和市政协的民主监督，定期向市人大及其常委会报告工作，向市政协通报情况、征询意见，认真听取各民主党派、工商联、无党派人士和人民团体的意见和建议。不断完善和切实落实政务公开制度，接受人民群众的监督。

四、进一步树立效能意识，完善管理求实效

新一届政府要坚持科学分工、分责，进一步理顺职责关系，通过工作职能和具体任务的合理分解，让市级政府、各区镇、各部门以及政府各级领导干部都切实承担起责任，发挥出积极主动性，促进协调配合，形成工作合力。要按照权责对应的原则，实施科学放权，特别是深入推进强镇（区）扩权改革，在人、财、事等方面赋予镇（区）更多自主权。进一步健全政府工作督查体系，加大督查力度，特别是抓好重大决策和重点工作跟踪督办。进一步完善政府绩效考评工作，落实各项问责制度。着力转变政府职能，强化服务型机关建设，稳妥推进事业单位分类改革，加快政府雇员制改革，通过政府购买服务等方式，降低行政成本，提供多元化、高品质的公共服务。

五、进一步树立廉政意识，健全制度重预防

新一届政府要继续深化源头治腐工作，不断完善惩防并举、注重预防的廉政体系，坚持用制度管权、管人、管事，确保权力规范运行、阳光运行。要加大审计、监察工作力度，加强对行政权力特别是重要岗位、重点领域行政权力的监督和制约，不允许政府部门“一把手”直接分管本单位工程项目和财务工作，进一步完善公共资源交易和公开招标拍卖制度，全面铺开公务卡改革，减少滋生腐败的机会。继续抓好廉洁从政教育，引导各级领导干部筑牢廉政思想基础。严肃查处各类违法违纪案件，坚决纠正损害群众利益的不正之风。

各位代表，国际旅游岛建设催人奋进，“十二五”蓝图激励人心。今后的五年，是充满机遇与挑战的五年，时代赋予我们光荣而神圣的使命。让我们更加紧密地团结在以胡锦涛同志为总书记的党中央周围，在市委的正确领导下，开拓创新，真抓实干，为建设更加卓越、更加和谐的三亚而努力奋斗！
